# Annotations

**Published:** 1907-12-28

**Authors:** 


### Annotations.

### Lord Kelvin.

The profession of medicine, associated as it is with science and scientific progress, must necessarily endeavour to take an adequate share in the tribute which is just now being rendered to the memory and work of the late Lord Kelvin. Though it cannot, perhaps, be said that this work had a very direct or intimate association with medicine, it still remains true that medical men in a very special manner are able to appreciate the record of achievement which placed Lord Kelvin in the foremost place both as a man of science and a practical inventor. Indeed, such a union of qualities may be said to make a special appeal to those whose work incessantly demands that theoretical speculation shall be linked to practical application. The combination just alluded to existed in a very unusual degree in the man whose much-regretted death has removed from British scientific men the active co-operation of their most distinguished leader. Also noteworthy in Lord Kelvin's career was the sustained capacity to welcome new ideas and to develop new researches and fresh trains of thought. It is recorded of him that on resigning his chair, after more than fifty years' service, as Professor of Natural Philosophy in the University of Glasgow, he entered his name as a matriculated student in order that he might continue to work in the laboratory as a Research Fellow. Little wonder that the University was proud to elect him as Chancellor and to cherish his name equally with the memory of Adam Smith and James Watt, among her most glorious traditions. Innumerable testimonies are being paid to his personal qualities not less than to his scientific greatness. Here it is not inappropriate to recall the fact that amidst his many distinctions he received the degree of M.D. from one of the German Universities, and, so the story goes, he once insisted on this title as justifying his order to an assistant to take an extra holiday! Lord Kelvin's name and work are secure for many generations, and his death is the close of a crowded life and a fruitful career.

### A Scale of Medical Fees.

In a recent action raised by a medical man to recover fees due for professional services, the contention of the defendant that the charges were excessive was urged on somewhat unusual grounds. The defence produced in Court a copy of a well-known almanack, in which, it appears, there is printed a statement professing to represent the sums charged by members of the medical profession for consultations, visits, and other professional attentions. The judge in the trial in question refused to allow this statement to be submitted as evidence, and a verdict was given for the plaintiff. Nevertheless it may be well that the existence of such an announcement in "Whitaker's Almanack" should be known to the medical profession, for this publication is widely consulted by members of the public. Personally we must confess that, though we have often to refer to our "Whitaker," we were quite unaware of the fact that in the almanack there appeared instructions on the subject of medical fees. The fact, however, is as stated at the trial, and the scale of charges is framed with very considerable detail. Upon what authority these charges rest is not stated, but appearing where they do it is not altogether surprising that some persons should regard them as officially authorised. This, of course, is a quite unwarranted conclusion, as every member of the profession is entitled to determine for himself what his fees shall be, though in case of a dispute the law will certainly take note of the "custom" practised by the profession in similar circumstances. So far as the actual figures given by "Whitaker" are concerned, they cannot be said to be seriously in error, considered as a general statement. But surely it is not reasonable to say that "all engaged in the profession are supposed to be equal in point of skill, and therefore entitled to charge alike, the tariff depending chiefly upon the residence of doctor or patient." Our purpose, however, is not to quarrel with the details of this published statement, but to draw attention to its existence and to repudiate its authority. The medical practitioner is free to frame his own scale of charges, and he usually does this with such help as he can get from the custom of his professional neighbours.

### Evidence and the Professions.

The value of evidence and the nature and worth of opinion are considerations which concern in a high degree both the medical and the legal professions. The authorities on the two sides are by no means in complete agreement, and the methods adopted in medicine are sometimes the subject of adverse comment by the representatives of law. In a recent address Professor Howard Marsh appears to have some sympathy with this suggestion, for he expresses the view that the importance of the scientific treatment of evidence has not been fully recognised in the medical profession. Everyone will agree with the learned professor that medical men are often imperfect both in their collection of evidence and in the inferences they draw from it. Yet with all deference we will venture to submit that the tasks which in this respect are set for the medical practitioner are often much more difficult than those which are presented to the sister profession. For it must be remembered that the lawyer, quite rightly from his point of view, has, by code or custom, a set of rules by which evidence is either included as relevant or excluded as irrelevant. The medical practitioner, on the other hand, cannot practise any such arbitrary method, but must be willing to collect and study everything which bears upon the issue presented to him. Take, for example, the question of "hearsay," to which the lawyer will give no heed, whilst the doctor has often to digest large supplies of it. There is another difference in the essential character of the work of the two professions in relation to evidence. In law the question is for the most part whether a certain proposition is proved by the evidence submitted, and an answer in the negative leaves the whole position open. But in medicine a diagnosis generally, or often, means a debate or the bearing of the available evidence upon several different propositions, and the urgency of the occasion compels a decision with practical and immediate consequences.

